# Effect of Tetrahydrofuran Extraction on Surface Functional Groups of Coking Coal and Its Wettability

**DOI:** 10.1155/2019/1285462

**Published:** 2019-06-26

**Authors:** Jian Yao, Huaijun Ji, Huazhang Lu, Tongtong Gao

**Affiliations:** School of Engineering and Technology, China University of Geosciences (Beijing), Beijing, China

## Abstract

Coking coal was extracted with tetrahydrofuran solvent using ultrasonic and microwave-assisted method at 50°C and atmospheric pressure. Wettability of raw coal and its residue (residual coal) was tested with capillary penetration method. The raw and residual coals were studied by Fourier transform infrared spectroscopy (FTIR) with curve-fitting analysis. The variation of main surface functional groups of coking coal before and after extraction and its effect on wettability were analyzed. The results were obtained as the following: after extraction with tetrahydrofuran, hydroxyl, ether oxygen, and carbonyl in the coal structure were dissolved, the content of hydrophilic functional groups reduced, and then the hydrophobicity of coal enhanced. At the same time, part of aliphatic hydrocarbon dissolved, the length of aliphatic chains (*I*_2_) decreased from 3.961 of raw coal to 3.636 of residual coal, the length of aliphatic chains became shorter, aliphatic CH_2_ side-chains decreased and aliphatic CH_3_ side-chains increased, and hydrophobic functional groups content increased. In the aromatic structure, four hydrogens per ring increased and two, three, and five hydrogens per ring decreased. Reduction of substitution functional groups and aliphatic hydrocarbon decreased with the side-chains breakage produce more active sites, which increases the degree of condensation of the aromatic ring (*I*_3_). The combined action of the decrease of the hydrophilic functional groups and the increase of the hydrophobic functional groups made the wettability of the coking coal become weak.

## 1. Introduction

The coal resources in China are rich, whereas in the process of exploitation, because of the high degree coalification of coking coal, the wettability is poor and it is difficult to be deposited [1], which causes the underground coal dust to be overlimit. After the coal dust reaches a certain concentration, there is a risk of explosion [2, 3]; on the other hand, long-term inhalation of coal dust will increase the incidence of pneumoconiosis and endanger workers' health [4]. At present, coal seam injection and water spray wet dust removal are mainly used in mining for dust control [5]. However, coal wettability is the key factor affecting application of these techniques.

Coal is a complex mixture of organic and inorganic minerals [6]; in the aromatic structure of organic macromolecules, there are also solvent-soluble organic micromolecules [7], which are mainly composed of aromatic compounds, aliphatic compounds, and oxidation compounds, accounting for 10%∼30% of organic minerals [8–10]. Zhou and Shi [11] measured Xinjiang Changyan coal's soluble mixture of solvent toluene/ethanol extraction by gas chromatography/mass spectrometry (GC/MS) and showed sixty-three organic compounds including aliphatic hydrocarbons, arenes, and organoheteratom compounds. Kunkun et al. [12] investigated major functional group changes of coking coal extracted from tetrahydrofuran (THF), carbon disulfide (CS_2_), and acetic acid (HAc) and found that fatty hydrocarbon and oxygenic functional groups in the proextracted coal decrease in different degrees. Ji et al. [13] studied the pore variation characteristics of coal after dissolution of organic micromolecules and considered solvent extraction exerted different effects on pores. Pores of gas coal expanded with reduced specific surface area, but pores of coking coal increased with increasing specific surface area. The dissolution of organic micromolecules in coal will inevitably change the coal's microstructure and affect the wettability of coal.

As an effective method to study the microstructure and chemical composition of coal, FTIR has been widely used in recent years [14–16]. Some scholars used infrared spectroscopy to study the assignment of functional groups in coal [17, 18] and to study the relationship with wettability. Ren et al. [19] conducted contact angle and infrared spectroscopy experiments on three kinds of coal samples before and after anionic surfactant sodium dodecyl sulfate (SDS) treatment and found that the change of wettability of coal depends on the change of surface functional groups, mainly by -OH and -COOH of hydrophilic groups. Weimin et al. [20] established the quantitative relationships between the coal dust wetting contact angle and the absorption intensity of surface functional groups and found that the surface's inorganic mineral functional groups, the oxygenated functional groups, and the organic molecular structures had great influences on coal dust wettability. Therefore, an in-depth understanding of the surface microstructure of coal is helpful to improve dust removal efficiency.

In the present research, coking coal was extracted with tetrahydrofuran (THF), and wettability of raw and residual coals was tested with capillary penetration method. FTIR was employed to analyze surface functional group changes of raw and residual coals. The effect of surface functional group variation of coking coal before and after extraction on wettability was analyzed. The research results provide a theoretical basis for understanding the mechanism of the influence of coal microstructure on its wettability and promoting coal mine dust control.

## 2. Experimental Section

### 2.1. Coal Sample

Coking coal collected from Linhuan Coal Mine was adopted as experimental sample. The coal sample was newly exposed to underground colliery, which was immediately loaded into the sealed bag and transported to the laboratory. After polishing and crushing coal samples, screening between 60∼100 mesh size (0.15∼0.25 mm) 200 g samples.  Table 1 lists proximate analysis results and THF extraction rate of coking coal.

### 2.2. THF Extraction Experiment

25 g coal sample and 250 ml of THF solvent were mixed and placed in the SL-SM100 ultrasonic processor and microwave extractor, set at 50°C, atmospheric pressure, extraction time 4 h, microwave power 400 W, ultrasonic power 270 W, working time 1 s, and interval 3 s. Repeat the experiment once and extract a total of 50 g coal sample. After the extraction was completed, extract liquid and residual coal were separated by vacuum suction filtration, residual coal was dried in a vacuum drying oven for 12 h at 80°C. The mass of residual coal was recorded and the extraction rate was calculated according to equation (1). The results are shown in Table 1.

(1) $$\begin{array}{c} E=\frac{\left(m_{1}-m_{2}\right)\times 100}{m}, \end{array}$$

where *m* is the mass of raw coal; *m*_1_ and *m*_2_ represent masses of filter paper with residual coal after drying and filter paper, respectively.

### 2.3. Wettability Experiment

**Figure 1:**
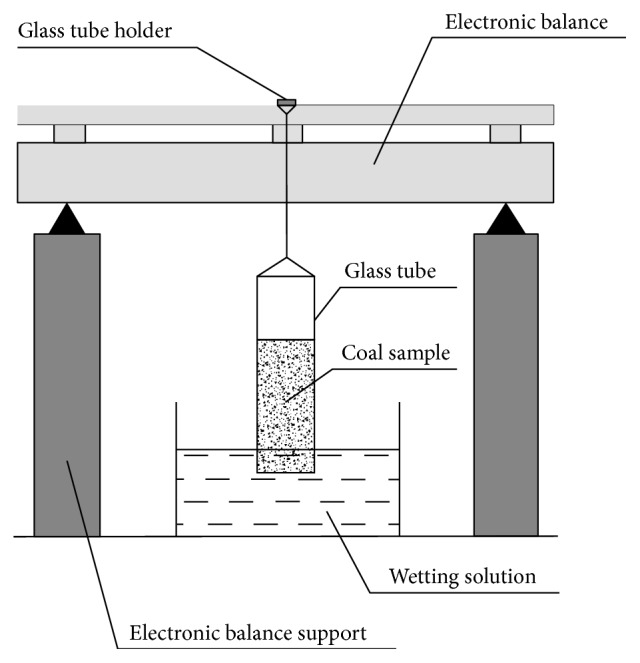
Schematic diagram of wettability experimental device.

According to capillary penetration, the schematic diagram of the wettability experimental device is shown in Figure 1. We weighed the 10 g coal sample, then placed in the glass tube, which is the inner diameter of 7 mm, height of 300 mm, and made of Perspex. The bottom of glass tube was sealed with filter paper and permeable tape, and then coal sample was compacted with glass rod. Finally, the bottom of glass tube was placed in distilled water and sodium dodecyl sulfate solution (SDS), respectively, used in experiments. The electronic balance was recorded every 10 minutes; the test time was 2 hours.

### 2.4. FTIR Measurements

Raw coal and residual coal were measured in FTIR; FTIR measurements were carried out using Nicolet iS10 FTIR spectrometer, the coal sample and KBr were mixed and ground at a mass ratio of 1 : 150. Coal samples were analyzed from the wavenumber range of 400–4,000 cm^−1^, at the resolution of 4 cm^−1^, with the collection of 32 scans per spectrum, at the signal-to-noise ratio of 50,000 : 1.

## 3. Results and Discussion

### 3.1. Wetting Characteristics of Raw Coals and Residual Coal

**Figure 2:**
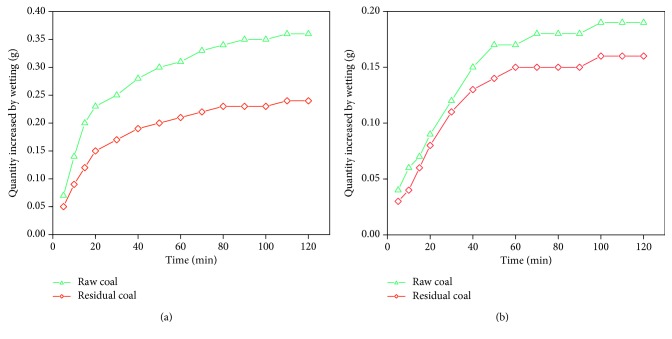
Wettability of raw and residual coals in different solution: (a) SDS solution; (b) deionized water.

Wetting experiment of raw coal and residual coals were conducted with 0.2% SDS and deionized water. The results are shown in Figure 2.

As shown in Figure 2, SDS solution exhibited superior wetting effects on raw and residual coal than deionized water; the wettability of raw coal was higher than residual coal in two solutions. In deionized water solution, the mass increase of raw coal wetting was 0.19 g, the residual coal was 0.16 g, and the decrease was 15.79%, while in SDS solution, the mass of wetting of raw coal was 0.36 g, the residual coal was 0.24 g, and the decrease was 33.33%. The results showed that after extraction with tetrahydrofuran, the wettability of coal became worse when part of organic micromolecules was dissolved.

### 3.2. Changes of Functional Groups before and after Extraction

**Figure 3:**
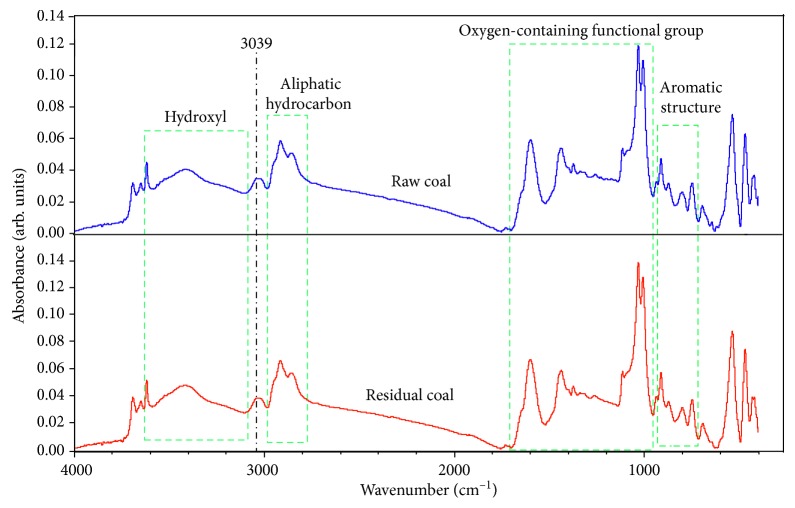
Infrared spectrum of raw coal and residual coals.

According to the literature on the attribution of functional groups in coal [17, 21, 22], the whole spectrum was divided into four parts, as indicated in Figure 3. A distinct absorption peak was caused by aromatic hydrogen at 3039 cm^−1^. Figure 3 shows that aromatic structure (700∼900 cm^−1^), oxygen-containing functional group (1000∼1800 cm^−1^), aliphatic hydrocarbon (2800∼3000 cm^−1^), and hydroxyl (3000∼3600 cm^−1^) had some differences between raw and residual coals. It is indicated that the surface functional groups were changed before and after tetrahydrofuran extraction.

#### 3.2.1. Hydroxyl Peak Fitting and Change Analysis

**Figure 4:**
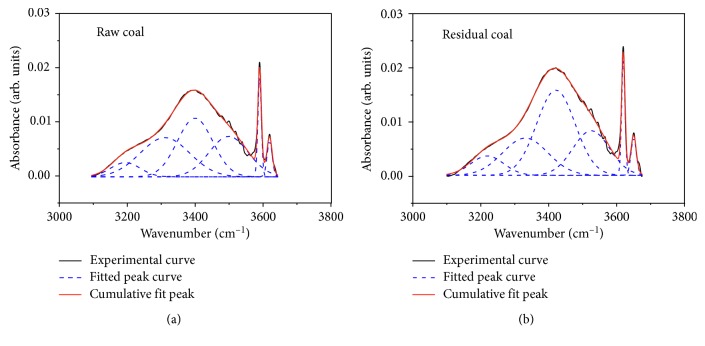
Curve-fitting FTIR spectrum of hydroxyl in raw and residual coals.

Hydroxyl was an important functional group that affects the wettability of coal and was also the main functional group that forms hydrogen bonds. According to the classification [23, 24], the spectrum of this region includes 5 kinds of hydroxyl, they were ring hydrogen near 3218 cm^−1^ zone, OH-OR near 3320 cm^−1^ zone, OH-OH near 3420 cm^−1^ zone, OH-*π* near 3525 cm^−1^ zone, and free hydroxyl near 3619 cm^−1^, and 3650 cm^−1^ zone. The five hydroxyl segments (3100∼3675 cm^−1^) were fitted with 6 Gaussians, and the fitting results are shown in Figure 4. As shown in Figure 4, there were two obvious peaks of raw and residual coals at 3619 cm^−1^ and 3650 cm^−1^. Most scholars attributed these two peaks to free hydroxyl group [23], and some scholars thought it was the influence of crystalline water in clay minerals [24, 25]. Based on the analysis of industrial analysis (Table 1) and the stretching vibration peak of ash at 1005 cm^−1^, it was concluded that the free hydroxyl in coal sample was caused by crystalline water in clay minerals.

According to the fitting results, the proportion of OH-OH absorption intensity between raw and residual coals was the largest, which was 31.78% and 41.92%, respectively. Secondly, the proportion of OH-OR absorption intensity and OH-*π* absorption intensity were 29.67% and 21.59% and 24.08% and 20.86%; the proportion of ring hydrogen and free hydroxyl was the smallest. After extraction with tetrahydrofuran, the absorption intensity of OH-OR and OH-*π* decreased to a certain extent, indicating that the dissolution of organic micromolecules in coal resulted in the destruction of OH-OR and OH-*π* hydrogen bonds.

#### 3.2.2. Aliphatic Hydrocarbon Peak Fitting and Change Analysis

**Figure 5:**
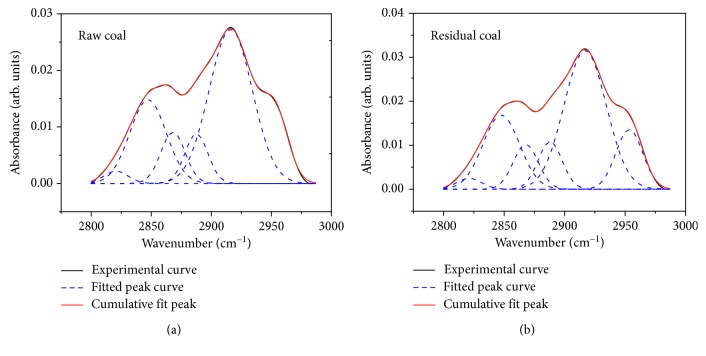
Curve-fitting FTIR spectrum of aliphatic hydrocarbons in raw and residual coals.

The structure of aliphatic hydrocarbon was observed in the 3000∼2800 cm^−1^ zone, and good fit was obtained with 6∼8 Gaussians in this region. The fitting curves of aliphatic hydrocarbon peaks of raw coal and residual coal are shown in Figure 5. As shown in Figure 5, two distinct peaks at 2850 cm^−1^ and 2920 cm^−1^ zones attributed to symmetric -CH_2_- stretching and asymmetric -CH_2_- stretching, respectively, were observed in raw and residual coals. In addition, there were symmetric -CH_3_ stretching vibration absorption peaks near 2870 cm^−1^ zone, asymmetric -CH_3_ stretching vibration absorption peaks near 2950 cm^−1^ zone, and methine C-H stretching vibration absorption peak near 2890 cm^−1^ zone. According to fitting results, the proportion of symmetric and asymmetric -CH_2_- stretching was dominated in aliphatic hydrocarbon of raw and residual coals, accounting for 23.74% and 23.49 and 47.32% and 46.06%, respectively. After extraction with tetrahydrofuran, the vibration absorption intensity of CH_2_ decreased, the vibration absorption intensity of CH_3_ and CH increased, and the structure of aliphatic hydrocarbons was changed.

#### 3.2.3. Oxygen-Containing Functional Group Peak Fitting and Change Analysis

**Figure 6:**
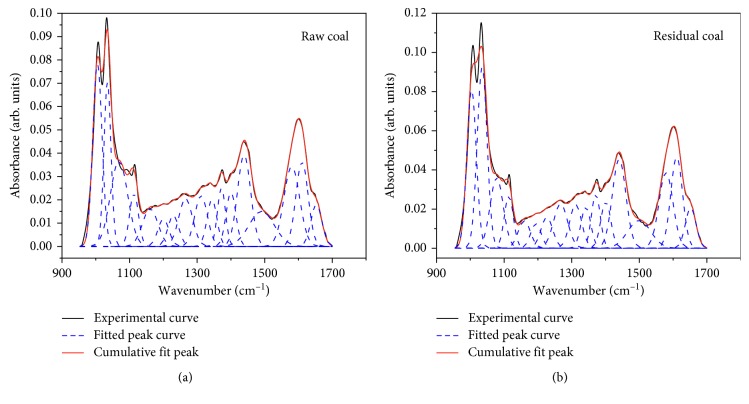
Curve-fitting FTIR spectrum of oxygen-containing functional group in raw and residual coals.

The spectrum of 1000∼1800 cm^−1^ zone was complex, mainly including oxygen-containing functional groups, such as ether oxygen, phenol hydroxyl, carbonyl, and carboxyl, as well as deformation vibration of CH_2_ and CH_3_ and aromatic C=C stretching vibration. Due to the high degree of metamorphism of coking coal, carboxyl at 1700 cm^−1^ zone was metamorphosed and no vibration peak appeared, so the influence of carboxyl group was not considered. The infrared spectrum 1000∼1700 cm^−1^ zone was fitted with 17 Gaussians. The fitting curve of oxygen-containing functional groups of raw and residual coals is shown in Figure 6. The 1100∼1340 cm^−1^ zone was mainly ether oxygen, phenol hydroxyl, and carbonyl stretching vibration, the vibrational peaks of asymmetric CH_3_- and CH_2_- near 1440 cm^−1^ and the vibrational peak of aromatic C=C at 1600 cm^−1^ were obvious and spike-like. After extraction with tetrahydrofuran, oxygen-containing functional groups in coal decreased, the content of ether oxygen decreased from 14.34% of raw coal to 11.15% of residual coal, and the phenolic hydroxyl decreased from 18.52% of raw coal to 16.81% of coal. The vibrational content of aromatic C=C was higher, and it decreased slightly after extraction, which indicates that the aromatic substance was stable and not easy to be destroyed.

#### 3.2.4. Aromatic Structure Peak Fitting and Change Analysis

**Figure 7:**
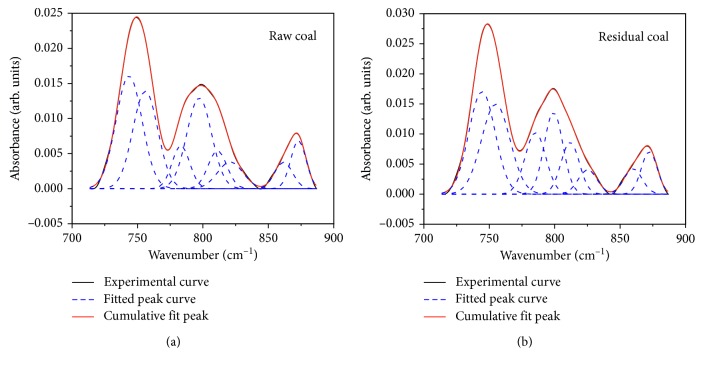
Curve-fitting FTIR spectrum of aromatic structure in raw and residual coals.

The aromatic structure was in the 700∼900 cm^−1^ zone of infrared spectrum, and it could be fitted with 6–8 Gaussians; the fitted result of raw and residual coals is shown in Figure 7. The absorption intensity of two adjacent hydrogens per ring at 750 cm^−1^ and three adjacent hydrogens per ring at 800 cm^−1^ were relatively large, indicating that two and three adjacent hydrogens per ring dominated the aromatic structure. According to the fitting results, after extraction with tetrahydrofuran, four adjacent hydrogens per ring increased, two, three, and five adjacent hydrogens per ring decreased, and then the substituted groups in aromatic structure decreased.

### 3.3. Analysis of the Effect of Surface Functional Group Changes on Wettability

Since the peak area of the infrared spectrum was less affected by the factors of the sample and the instrument, the peak area obtained by fitted was used for quantitative analysis, and four infrared spectral parameters [15, 26] were selected to analyze the influence of wettability, and the results are shown in Table 2.

(2) $$\begin{array}{c} O_{\text{AL}}=A_{1100-1340}, \end{array}$$

where *A*_1100−1340_ is the integrated content of hydroxyl, ether oxygen, and carbonyl functional groups.

The parameter *I*_1_ is an index of relative content of aliphatic structural functional groups. Equation (3) was adopted for the estimation of *I*_1_:

(3) $$\begin{array}{c} I_{1}=\frac{A_{2800-3000}}{A_{1600}}, \end{array}$$

where *A*_2800−3000_ is the integrated area of 2800–3000 cm^−1^; it is used to estimate the total aliphatic CH content (CH_3_, CH_2_, and CH), and *A*_1600_ is attributed to the content of the aromatic C=C stretching at 1600 cm^−1^.

The parameter *I*_2_ corresponds to the length of aliphatic chains and the degree of branching aliphatic side-chains. The higher value of *I*_2_ represents the longer aliphatic chains and the fewer side-chains.

(4) $$\begin{array}{c} I_{2}=\frac{{\text{CH}}_{2}}{{\text{CH}}_{3}}=\frac{A_{2916}}{A_{2954}}, \end{array}$$

where *A*_2916_ and *A*_2954_ represent the content of asymmetric CH_2_ and CH_3_ stretching, respectively.

The parameter *I*_3_ represents the degree of condensation of aromatic rings. The higher value of *I*_3_ corresponds to the greater degree of condensation of aromatic rings.

(5) $$\begin{array}{c} I_{3}=\frac{A_{700-900}}{A_{1600}}, \end{array}$$

where  *A*_700−900_ is the content of the aromatic hydrogen.

As known from Table 2, hydrophilic oxygen-containing functional groups and hydrophobic aliphatic and aromatic structures had changed with the extraction of tetrahydrofuran. The specific analysis was as follows:

#### 3.3.1. Effect of Oxygen-Containing Functional Groups Changes on Wettability

The oxygen-containing functional groups in coal reduced the hydrophobicity of coal and enhanced the wettability [27]; that is, the greater the content of oxygen-containing functional groups, the stronger the wettability of coal. Japanese scholar Murata's [28] study showed that carboxyl in oxygen-containing functional groups had the greatest influence on the wettability of coal, followed by hydroxyl. After extraction with tetrahydrofuran, the hydrophilic functional groups of hydroxyl, ether oxygen, and carbonyl in coal were fallen from the coal. The value of *O*_AL_ was reduced from 5.502 of raw coal to 5.163 of residual coal, which enhances the hydrophobicity of coal.

#### 3.3.2. Effects of Aliphatic Structure Changes on Wettability

After extraction, the ratio of *I*_1_ decreased and part of the aliphatic hydrocarbon was dissolved. The vibration absorption intensity of CH_2_ decreased, and the vibration absorption intensity of CH_3_ and CH increased; the ratio of *I*_2_ decreases from 3.961 of raw coal to 3.636 of residual coal. The length of aliphatic chains became shorter, the methylene side-chains were broken and reduced, the methyl side-chains were increased, and the content of hydrophobic functional groups increased, which enhances the hydrophobicity of coal.

#### 3.3.3. Effect of Aromatic Structure Change on Wettability

The aromatic structure of coking coal was destroyed by tetrahydrofuran extraction, four adjacent hydrogens per ring increased, and two, three, and five adjacent hydrogens per ring decreased. The number of substituent groups in aromatic structure was reduced, and the addition of aliphatic hydrocarbon and side-chains ruptures produced more active sites. The two factors together increase the degree of condensation of the aromatic ring (*I*_3_) and enhance the hydrophobicity of the coal.

The hydroxyl, ether oxygen, and carbonyl hydrophilic functional groups decreased, and the hydrophilicity of coal weakened; part of the aliphatic hydrocarbon was dissolved, the side-chains of methylene were broken down, methyl side-chains were increased, the degree of condensation of aromatic rings was increased, and the hydrophobicity of coal was enhanced. The combined effect of reduced hydrophilic group content and increased hydrophobic group content weakened the wettability of the coking coal after extraction.

## 4. Conclusions

This paper used tetrahydrofuran solvent to extract coking coal and the wettability, and infrared spectrum experiments of raw and residual coals were carried out. The changes of surface functional groups of coal sample before and after extraction and their influence on wettability were discussed. The conclusions were as follows:

1. Oxygen in coking coal existed in the form of hydroxyl, ether oxygen, carbonyl, and other functional groups. After extraction with tetrahydrofuran, oxygen-containing functional groups of hydroxyl, ether oxygen, and carbonyl were dissolved, the content of hydrophilic groups was reduced, and the hydrophobicity of coal was enhanced.
2. After extraction of tetrahydrofuran, part of the aliphatic hydrocarbon dissolved, the length of aliphatic chains became shorter, the methylene side-chains were broken and reduced, the methyl side-chains increased, and the content of hydrophobic functional groups increased, which enhances the hydrophobicity of coal.
3. After extraction, four adjacent hydrogens per ring increased, and two, three, and five adjacent hydrogens per ring decreased. The substituted groups in the aromatic structure decreased, and the addition of aliphatic hydrocarbon and side-chains ruptures produced more active sites, the two factors together increase the degree of condensation of the aromatic ring and enhance the hydrophobicity of the coal.
4. After the dissolution of some organic micromolecules in the coal, the combined effect of reduced hydrophilic group content and increased hydrophobic group content weakened the wettability of the coking coal after extraction.

## Figures and Tables

**Table 1:** Industrial analysis and extraction rate of coal samples (THF).

Coal sample	Coal type	Proximate analysis (%)				*E* % (THF)
		*M*_ad_	*A*_d_	*V*_daf_	FC_d_	

LX	Coking coal	0.68	13.13	29.17	61.11	1.320

daf, dry ash free; *M*_ad_, moisture (air-dried base); *A*_d_, ash (dry base); *V*_daf_, volatile matter (dry ash free); FC_d_, dry fixed carbon (dry base).

**Table 2:** Infrared spectral parameters of raw and residual coals.

Coal sample	*O*_AL_	*I*_1_	*I*_2_	*I*_3_
Raw coal	5.502	0.741	3.961	0.371
Residual coal	5.163	0.712	3.636	0.462

## Data Availability

The data used to support the findings of this study are available from the corresponding author upon request.
